# Dynamic Mechanical Behavior of Fiber-Reinforced Seawater Coral Mortars

**DOI:** 10.3390/ma13010118

**Published:** 2019-12-26

**Authors:** Wu-Jian Long, Jiangsong Tang, Hao-Dao Li, Yaocheng Wang, Qi-Ling Luo

**Affiliations:** Guangdong Provincial Key Laboratory of Durability for Marine Civil Engineering, Shenzhen Durability Center for Civil Engineering, College of Civil and Transportation Engineering, Shenzhen University, Shenzhen 518060, China; longwj@szu.edu.cn (W.-J.L.); tangjiangsong2018@email.szu.edu.cn (J.T.); lihaodao2017@email.szu.edu.cn (H.-D.L.); wangyc@szu.edu.cn (Y.W.)

**Keywords:** coral aggregate, PVA fiber, seawater coral mortar, dynamic mechanical behavior

## Abstract

Coral aggregate has been widely used for island construction because of its local availability. However, the addition of coral aggregate exaggerates the brittle nature of cement-based materials under dynamic loading. In this study, polyvinyl alcohol (PVA) fiber was used to improve dynamic mechanical behavior of seawater coral mortars (SCMs). The effects of coral aggregate and PVA fiber on the workability, static mechanical strengths, and dynamic mechanical behavior of fiber-reinforced SCMs were investigated. Results showed that the workability of the SCM decreased with increasing coral aggregate replacement rate and PVA fiber content. Mechanical strengths of the SCM increased with increasing PVA fiber content, but decreased with increasing coral aggregate replacement rate. Dynamic mechanical behavior at varying coral aggregate replacement rates was analyzed by combining dynamic mechanical analysis and micro-scale elastic modulus experiment. With increasing coral aggregate replacement rate, the storage modulus, loss factor, and elastic modulus of the interfacial transition zone in the SCM decreased. Nevertheless, with the incorporation of PVA fibers (1 vol.%), the storage modulus and loss factor were improved dramatically by 151.9 and 73.3%, respectively, compared with the reference group. Therefore, fiber-reinforced coral mortars have a great potential for use in island construction, owing to the excellent anti-vibrational performance.

## 1. Introduction

Sustainable island construction remains a challenge because transportation for large amounts of aggregates and fresh water from land to ocean construction sites is uneconomical. Therefore, the reasonable exploitation of marine resources (e.g., dead coral reefs and seawater) is significant. A recent *Nature* report showed that raising sea surface temperatures have increased the frequency and intensity of coral bleaching events. It also predicted that most coral reefs in tropical oceans would die within the next 80 years [1]. Such a huge amount of dead coral reefs can be expected to be used as locally available aggregates in concrete, to solve the transportation problem.

A coral reef, the main mineral components of which are aragonite and high-magnesium calcite, is made up of thin layers of over 95% calcium carbonate [2]. Compared with natural aggregates, coral reef aggregates have rough surface, irregular shape, and high porosity [3]. As early as the Second World War, corals have been used as a concrete ingredient to build airports, roads, and architecture in Pacific Atolls [4]. In 1991, Rick [5] investigated three coral concrete structures at Bikini Atoll. Results showed that the strength of coral concrete increased by 55–60% after 11 years, confirming that high-quality coral concrete exhibits long-term stability. Lyu et al. [6] found that coral aggregates can absorb more water than natural aggregates due to the major difference between the surface morphology and internal porosity. Guo et al. [7] reported that the compressive and flexural strengths of coral concrete are much lower than those of ordinary concrete. However, adding supplementary cementitious materials can solve this problem. Cheng et al. [8] found that the 28-day mechanical strength of coral concrete is higher than that of ordinary concrete, owing to the incorporation of metakaolin and blast furnace slag. Furthermore, the workability, volume stability, and durability of coral concrete has also been studied [9,10,11,12,13]. Xu et al. [9] found that the workability of concrete gradually decreased with an increase in the replacement rate of coral reef sand; when the replacement rate was 100%, the slump was reduced to about 80 mm. Liu et al. [10] used pre-wetted coral aggregates to prepare ultra-high performance concrete (UHPC) and studied their effect on autogenous shrinkage. Results showed that the addition of saturated coral aggregates into UHPC can help decrease the autogenous shrinkage, regardless of the aggregate content. Cheng et al. [11] revealed that river sand concrete exhibits lower impermeability than coral sand concrete at all tested ages. In addition, they found that adding metakaolin and blast furnace slag can help reduce the drying shrinkage of coral sand concrete. According to previous studies, coral aggregate concrete can be used as a building material.

Dynamic mechanical properties refer to the stress–strain curve of a material under dynamic loading. Notably, marine concrete structures need to withstand different types of dynamic loads such as from waves, typhoons, and earthquakes. They undergo severe corrosion due to waves, leading to a deterioration in the vibrational capacity during their long-term service period [14]. Long-term dynamic loads have a negative impact on the service life of marine concrete structures, particularly when exposed to salt efflorescence and transverse cracking [15]. Dynamic mechanical properties of the mortars were assessed by measuring their storage modulus and loss factors. A higher loss factor suggests a greater phase displacement between the given stress and measured strain of the seawater coral mortar (SCM) specimens, implying an improved damping property. Recently, Long et al. reported that the addition of graphene oxide to cement paste can enhance the dynamic mechanical properties, and clarified its reinforcement mechanism [16]. The dynamic mechanical properties of alkali-activated slag mortar with standard curing were higher than the natural curing at 28 days, and waste rubber tires used as a substitute for fine aggregates can enhance the dynamic mechanical properties of alkali-activated slag mortar, explained by series and parallel model mechanism [15,17]. However, studies about dynamic mechanical properties of coral aggregate cement composites remain scarce.

Reinforcing concrete with fibers is a promising method for improving the dynamic mechanical properties of concrete structures. The improved toughness, ductility, and energy consumption of fiber-reinforced concrete can be attributed to the high energy absorption capacity of the strain-hardening cementitious composites with fibers [18,19]. The extensive plastic deformation of fibers during protrusion contributes considerably to the energy dissipation of cementitious composites under impact loading [19]. Zhao et al. [20] reported that fibers in the matrix improve the energy consumption of concrete and change the failure mode from brittle to ductile. Compared with other fibers such as polypropylene fiber and polyethylene fiber, polyvinyl alcohol (PVA) fiber has high strength and elastic modulus, and it has good durability and interfacial bonding strength with cement matrix [21]. Thong et al. [21] found that PVA fiber as a reinforcing material can help significantly improve the impact resistance. However, the application of PVA fiber to improve the dynamic mechanical properties of SCM is still limited.

Therefore, the aim of this study was to investigate the workability, static mechanical properties, and dynamic mechanical behavior of fiber-reinforced seawater coral mortar (SCM) by varying the aggregate replacement rate (0, 30, 60, and 100 wt.%) and PVA fiber content (0, 0.25, 0.5, and 1.0 vol.%). Dynamic mechanical properties (i.e., storage modulus and loss factor) of the SCM under varying temperatures and frequencies were investigated. By using the statistical nanoindentation technique (SNT), we quantified the microscopic elastic modulus of the interfacial transition zone (ITZ), while the relationship between the storage modulus and the ITZ elastic modulus was analyzed. Furthermore, the failure behavior of PVA fiber in the SCM and the effect of the fiber on energy dissipation were evaluated by scanning electron microscopy (SEM) for revealing the reinforcing mechanism from its addition. In addition, we also proposed the mechanism of temperature change on the dynamic mechanical properties of the SCM. The findings of this study help to not only understand the behavior of marine structures under dynamic loading, but also demonstrate the application potential of fiber-reinforced SCM with excellent dynamic mechanical behavior.

## 2. Materials and Methods 

### 2.1. Raw Materials

Ordinary Portland cement (PI 42.5R) and fly ash (a kind of supplementary cementitious material) used in this study conformed to the requirements of the Chinese Standard GB175 [22] and GB/T1596 [23], respectively. Table 1 lists the chemical composition of the cement and fly ash. Sea water was used as the mixing water, taken from the South China Sea. The major ionic compositions were determined by inductive coupled plasma mass spectrometry (ICP–MS, Thermo Fisher Scientific, Shenzhen, China), as listed in Table 2.

**Figure 1:**
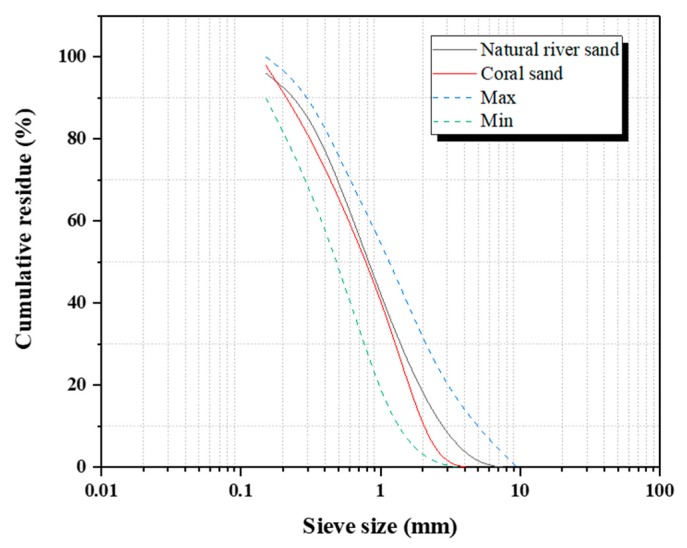
Particle size distribution of the natural river sand and coral sand.

**Figure 2:**
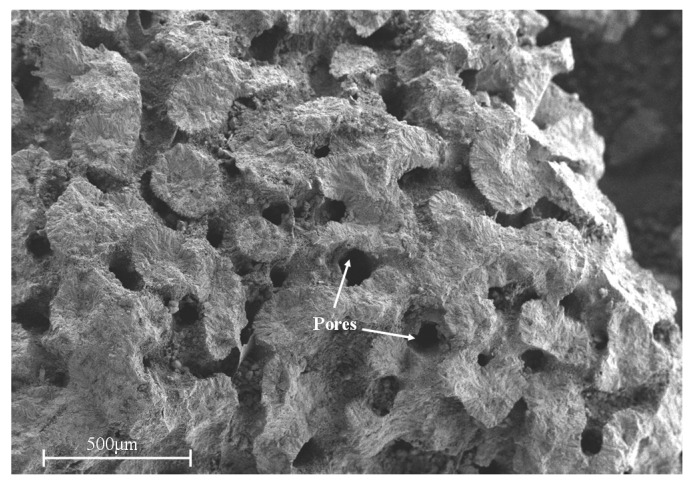
Microstructural morphology of coral sand.

The natural river sand used in this study was obtained from Xiamen ISO Standard Co., Ltd (Xiamen, China); the test results were consistent with the Chinese standards [24]. Coral reef samples were obtained from the South China Sea. The samples were crushed using a jaw breaker to produce coral aggregates, which passed meshes with sieve sizes between 0.075 and 4.75 mm. Figure 1 shows the particle size distribution of the natural river sand and coral sand. Figure 2 shows the morphology of the coral sand aggregates obtained using SEM, and the surface of the coral sand was porous, rough, and angular. Table 3 lists the basic physical properties of the coral sand and natural river sand, which were obtained from the experiment. 

PVA fiber with a length of 12 mm and a tensile strength of 1620 MPa was acquired from Kuraray, Tokyo, Japan. PVA fibers were surface-treated with surface active agent (i.e., polyethylene glycol type) in the wet/dry-jet wet spinning production process. The fiber surface was smooth, and the fibers were held together tightly. Table 4 lists the physical properties of the PVA fiber. To improve the workability of the freshly prepared mortar, a polycarboxylate-based high-range water reducing admixture (HRWRA) was employed in this study, consistent with the requirements of the JG/T223 Standard [25]. 

### 2.2. Mortar Preparation

SCM samples with different mixture proportions (Table 5) were prepared. The mortar was mixed using a high-shear mixer and then cast into a circular truncated cone for the slump flow expansion measurement. Custom-made cuboid molds with a size of 40 × 40 × 160 mm^3^ and 40 × 40 × 80 mm^3^ were used for compressive strengths testing and flexural strengths testing, and cubic molds with a size of 20 × 20 × 20 mm^3^ were used for dynamic mechanical property testing. After demolding, the samples were cured in a standard curing room maintained at a temperature of 20 °C and a relative humidity of 95% for two different curing periods: 7 days and 28 days. The mixing sequence for the SCM is as follow. First, sand, cement, fly ash, and PVA fiber were mixed and stirred for 1 min in a high-shear mixer to uniformly disperse the PVA fiber. Thereafter, HRWRA and seawater were blended and stirred using a glass rod for 30 s and placed in the high-shear mixer. Finally, the materials were mixed at a low speed for 1 min and then at a high speed for another 1 min.

### 2.3. Test Methods

#### 2.3.1. Fluidity Test

The effects of aggregate replacement rate and PVA fiber content on the slump flow expansion were tested, with conformance to GB/T2419-2005 [26]. The cone used for this test had a top diameter of 36 mm, a bottom diameter of 60 mm, and a height of 60 mm.

#### 2.3.2. Flexural and Compressive Strengths Testing

To investigate the effects of aggregate replacement rate and PVA fiber content on the mechanical properties of the specimens, both flexural and compressive strengths experiments were carried out. Each group of three samples was examined at 7 and 28 days and the strengths were determined by the average value. The loading rates were 50 N/s and 2.4 KN/s with conformance to GB/T17671-1999 [27]. In particular, the support distance in terms of the flexural strength test was 100 mm.

#### 2.3.3. Dynamic Mechanical Analysis

Dynamic mechanical analysis (DMA) was used to characterize the dynamic mechanical properties of the samples by measuring their storage modulus and loss factors. According to the fundamental principle of time–temperature superposition, the DMA was performed at a heating rate of 5 °C/min, a vibration frequency range of 0.5–2 Hz, and a temperature range of −30 to 50 °C. The maximum dynamic force applied to the specimens was 80 N, and the static force was −100 N. To avoid the effect of void water, the samples were dried in a vacuum desiccator at a temperature of 40 °C until the weight remained constant. After 28 days of curing, DMA tests were conducted, and the experimental results were recorded through a data acquisition system.

#### 2.3.4. Nanoindentation Mechanical Analysis

For the nanoindentation test, we used an optical nanoindenter (Hysitron TI-950 nanoindenter, Bruker, Shenzhen, China) with a Berkovich probe. The 20-mm cube samples were polished for the nanoindentation experiments. We referred to the study conducted by Long et al. for details pertaining to the loading, holding, and rapid unloading phases [28]. The maximum indentation force was 600 μN, and the indentation depth was less than 300 nm. The indentations were carried out in a grid with an area of approximately 90 × 90 μm^2^ and a separation distance of 10 μm. The 100 indentation points on the 90 × 90 μm^2^ material were considered to be representative of the cement samples under the optical microscope. The raw data for each indentation were examined, and the abnormal load–penetration curve of the polished surface was eliminated [29]. The main cause of the abnormal load–penetration curve is the unstable contact between the tip and the surface of the specimens or the sudden jump in the initial loading. The load–depth curves were recorded to obtain the micromechanical properties of the SCM. To analyze the experiment data, the analytical method proposed by Oliver and Pharr was used to calculate the elastic modulus from the load–penetration curve [28].

## 3. Results and Discussion

### 3.1. Flow Table

**Figure 3:**
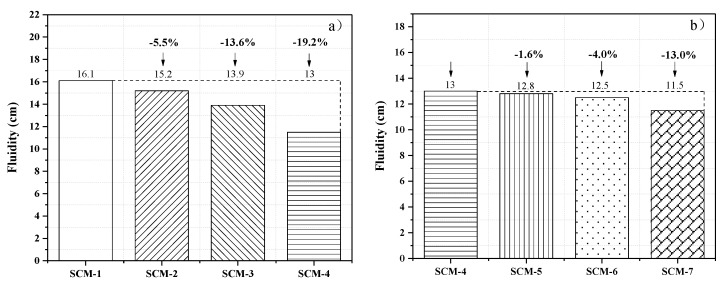
Slump flow diameters of seawater coral mortar (SCM) samples at varying (**a**) coral sand replacement rates and (**b**) PVA fiber contents.

The slump flow expansion of the mortar was tested by conducting mini-slump tests. Figure 3a,b shows the slump flow diameters measured at varying aggregate replacement rates and fiber contents. Figure 3a shows that with the increase in the coral sand replacement rate, the slump flow expansions of the SCM samples decrease. At a constant water–cement ratio of 0.4, the slump flow expansions of the samples (SCM-1–7) are 16.1, 15.2, 13.9, 13, 12.8, 12.5, and 11.5 cm, respectively. Compared with the reference sample SCM-1, the workability of SCM-2, SCM-3, and SCM-4 is reduced by 5.5, 13.6, and 19.2%, respectively. This is because the surface of coral aggregates is rougher than that of standard sand, resulting in an increase in the friction between the cement matrix and the aggregates. In addition, coral sand probably soaked up a high amount of water and thus the slump flow expansion of the mixture was reduced. 

Figure 3b shows that the slump flow expansion of the samples slightly decreases with the increase in the PVA fiber content. This is because the hydrophilic PVA fiber consumes more water in the mixing stage. In addition, as the proportion of fiber increases, more cement paste is consumed to cover the fibers, leading to a smaller amount of paste for the slump flow [30].

### 3.2. Compressive and Flexural Strengths

**Figure 4:**
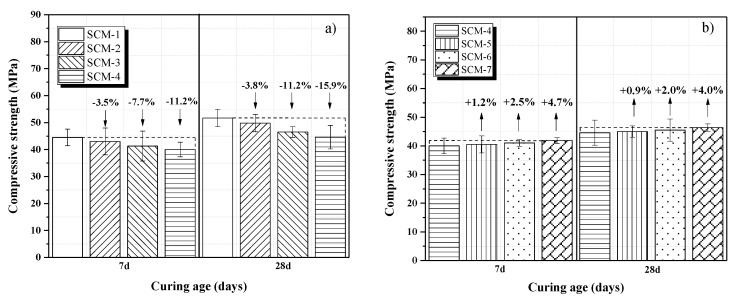
Results of compressive strength tests of SCM samples at varying (**a**) coral sand replacement rates and (**b**) PVA fiber contents.

Figure 4a,b shows the compressive strengths of the samples. The compressive strength increases with increasing curing age. However, as the coral aggregate replacement rates increases, there is a negative effect on the compressive strengths development of SCM from day 7 to day 28. With curing age from 7 to 28 days, the compressive strength of the reference sample (SCM-1) increases by 16.17%, whereas that of SCM-4 only increases by 11.5%. This is because the coral sand absorbs water in the early stage of mixing [31,32]. In the process of cement hydration, the release of water from the coral sand promotes the hydration of the cement around the coral sand aggregates, thus leading to an early increase in the compressive strength of the coral sand mortar. Figure 4a shows that the compressive strength of the SCM samples slightly decreases with increasing coral aggregate replacement rate. In particular, the compressive strengths of SCM-2, SCM-3, and SCM-4 hardened for 28 days are lower than that of the reference sample (SCM-1) by approximately 3.8, 11.2, and 15.9%, respectively. This can be attributed to the difference in the aggregate strengths between coral sand and natural river sand, and this result is consistent with a previous study result [33]. Figure 4b shows that the compressive strengths of the samples SCM-4–7 (with fiber contents of 0, 0.25, 0.5, and 1%) at 28 days are 44.6, 45, 45.5, and 46.4 MPa, respectively. Clearly, the addition of PVA fiber has no effect on the compressive strength of the samples. According to Li et al.’s research, short fibers can increase the compressive strength of the matrix. However, containing longer PVA fibers makes the cement matrix more difficult to vibration and consolidation, which has no positive effect on the increase in compressive strength [34].

**Figure 5:**
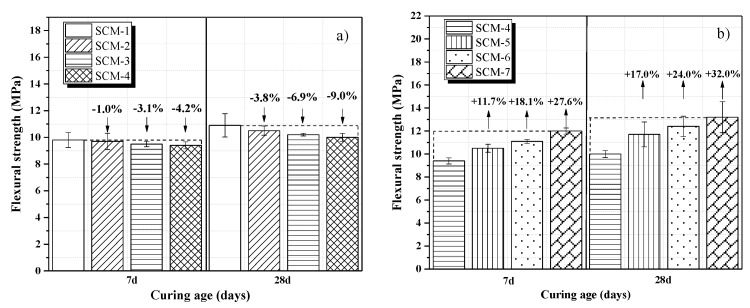
Results of flexural strength tests of SCM samples at varying (**a**) coral sand replacement rates and (**b**) PVA fiber contents.

Figure 5a,b shows the flexural strengths of the samples. Figure 5a shows that the addition of coral sand has a negative effect on the mortar flexural strength, and the flexural strength of the samples is minimum compared to the reference sample SCM-1 when the replacement rate of coral sand (SCM-4) is 100%. The early flexural strength of SCM develops rapidly, and the flexural strength of the 7-day and 28-day curing age has little effect. Figure 5a shows that the flexural strength decreases when the coral aggregate is added at 28-day. In Figure 5b, compared with SCM-4, the flexural strengths of SCM-5, SCM-6, and SCM-7 at 28 days increase by 17, 24, and 32%, respectively. The addition of PVA fiber significantly increases the flexural strength of the samples. As the fiber content rises, brittle failure of mortar changes to ductile failure under dynamic loads [35]. This is because the fibers play a bridging effect, whereby the occurrence and development of microcracks is controlled, thus increasing the flexural strength [36]. In particular, according to Pakravan et al.’s research, polymer fibers are less susceptible to the effects of high alkali environment and salt environment [37].

### 3.3. Dynamic Mechanical Behavior

Dynamic mechanical behavior of the mortars was characterized in terms of the loss factor and storage modulus. A higher loss factor indicates a higher phase displacement between the given stress and the measured strain, and therefore an improvement in the damping performance [17]. The storage modulus of the mortar is related to the stiffness and brittleness and is used to characterize the elastic behavior of mortars. The higher the storage modulus, the lower the deformation of the material under a given load.

#### 3.3.1. Influence of Aggregates

**Figure 6:**
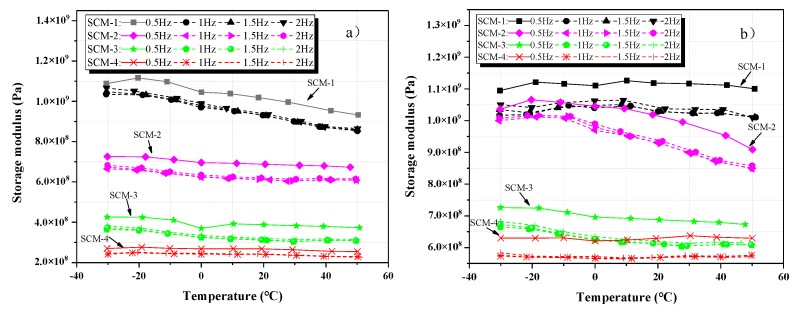
Relationship between the storage modulus and temperature of SCM-1, SCM-2, SCM-3, and SCM-4 at varying frequencies: (**a**) 7 days; (**b**) 28 days.

Figure 6a,b shows the relationship between the storage modulus and the temperature of SCM-1, SCM-2, SCM-3, and SCM-4 aged for 7 and 28 days at various frequencies. At the same temperature, the storage modulus of the samples is optimal at 0.5 Hz. However, the variation trends in the storage modulus at frequencies of 1, 1.5, and 2 Hz are similar. Comparing Figure 6a,b, we find that the storage moduli of SCM-1–4 aged for 28 days are higher than those of SCM-1–4 aged for 7 days, probably because of the higher degree of hydration of the cement matrix and the denser interface between the aggregate and the matrix. 

**Figure 7:**
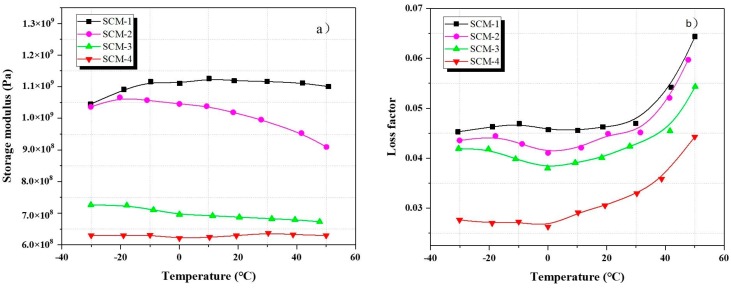
Samples under varying coral sand replacement rates and temperature for frequency of 0.5 Hz at 28 days: (**a**) Storage modulus; (**b**) loss factor.

Figure 7a shows the relationship between the storage modulus and the temperature of the samples aged for 28 days at 0.5 Hz; the addition of coral sand has a negative effect on the mortar storage modulus, and the storage modulus of the samples is minimum when the replacement rate of coral sand (SCM-4) is 100%. This is probably because the ITZ of the coral aggregates has a lower elastic modulus than river sand. This study clarified the mechanism later. Figure 7b shows the loss factors of the samples SCM-1, SCM-1, SCM-3, and SCM-4 aged for 28 days at 0.5 Hz as a function of the temperature. It should be noted that the trend of the relationship between temperature and loss factor is consistent. The loss factor decreases with increasing coral content. This is probably because the microcracks formed between the river sand and the cement matrix can convert energy into mechanical energy via the vibration of the aggregates, thus promoting energy consumption.

#### 3.3.2. Influence of PVA Fibers

**Figure 8:**
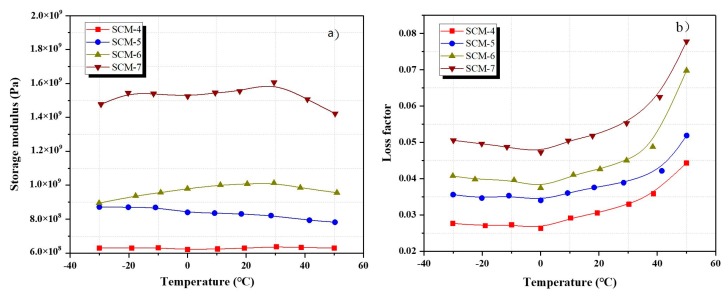
Dynamic mechanical analysis (DMA) analysis of samples under varying PVA fiber content and temperature for frequency of 0.5 Hz at 28 days: (**a**) Storage modulus; (**b**) loss factor.

Figure 8a shows the storage modulus of the SCM-4, SCM-5, SCM-6, and SCM-7 samples aged for 28 days. The storage modulus of the samples without PVA fiber (SCM-4) is lower than those of the PVA fiber-reinforced samples (SCM-5, SCM-6, and SCM-7) regardless of the temperature. The storage modulus of SCM-5, SCM-6, and SCM-7 aged at 28 days increase by 28.8, 58.9, and 151.9%, respectively, with the increase in the PVA fiber content, compared with that of SCM-4. This is because the fibers hinder the further development of cracks and maintain the toughness of the cement matrix under dynamic loads. It also can be noted that adding 0.25 and 0.5 wt.% PVA fiber has no significant effect on the enhancement of storage modulus, but adding 1.0 wt.% PVA fiber, the storage modulus of the SCM can be significantly enhanced.

The relationship between the loss factor and the temperature of the specimens aged for 28 days shows similar variation trends under different vibration frequencies (0.5, 1, 1.5, and 2 Hz). According to the experimental results, the storage modulus of the SCM is maximum at a frequency of 0.5 Hz, which then decreases with increasing frequency. As shown in Figure 8b, the loss factors of SCM-5–7 are higher than that of SCM-4 in the temperature range. The loss factors of the mortars containing 0.25, 0.5, and 1% (by volume) PVA fiber measured at a frequency of 0.5 Hz increase by 20.0, 40.0, and 73.3%, respectively, compared with that of the reference sample SCM-4. With the increase in the PVA fiber content, the loss factor increases. Thus, the addition of PVA fiber has a positive effect on the loss factor. In fact, there are multiple interfaces between the PVA fiber and the cement matrix, and the fiber inhibits stress concentration [16]. In the mortar containing PVA fiber under the dynamic loads, the breakage of PVA fiber consumes part of the energy in the form of mechanical energy. On the other hand, the thermal energy generated by the slip between the PVA fiber and the cement matrix also consumes a portion of mechanical energy. Therefore, the addition of PVA fiber to the mortar increase the value of loss factor because PVA fiber increases part of the energy dissipation.

### 3.4. Influence of DMA Temperature Changes on Loss Factor of Mortar

The loss factor of the specimens was measured under varying temperatures. Figure 7b and Figure 8b show the variation in the loss factor of the samples cured for 28 days in the temperature range of −30 to 50 °C and at a frequency of 0.5 Hz. The trend in the loss factor at different temperatures is roughly the same, with the loss factor being minimum at approximately −30 °C. There is no significant change in the loss factor between −30 and 0 °C. However, the loss factor increases rapidly with increasing temperature after 0 °C.

Marine mortar is a porous composite and is affected by temperature changes. When the internal temperature of the mortar is lower than 0 °C, the water in the capillary pores gradually freezes. When the water in the capillary pores is frozen, some of the internal pores in the mortar are filled with ice. According to Liu et al. [38], the pressure between the pore walls due to temperature contraction and the ice lead to excessive stress in the mortar. The excessive stress accelerates the destruction of the microstructure of the mortar. The loss factor of the marine mortar containing PVA fiber was higher than that of the reference sample SCM-4 at −30 °C, attributed to the bridging effect of the fiber that reduces the destruction of the microstructure.

### 3.5. Mechanical Properties of Interfacial Transition Zone

Nanoindentation tests have some drawbacks when it comes to analyzing the results, because the mortar is a porous material with a pore size usually greater than the diameter of the indenter. Nevertheless, the microstructure of the mortar can be analyzed based on the nanoindentation results. The elastic moduli of the different hydration products, anhydrous cement particles, and aggregates are as follows [39]: Porosity: 0–8 GPa; calcium silicate hydrates and ettringite crystals: 8–30 GPa; calcium hydroxide crystals: 30–50 GPa; unhydrated cement particles, natural aggregates: ≥50 GPa.

**Figure 9:**
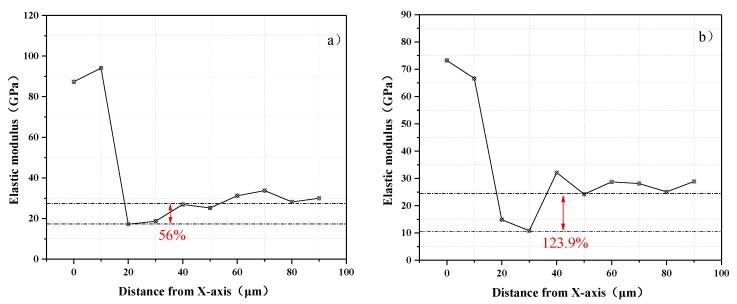
Results of nanoindentation experiments: (**a**) SCM-1; (**b**) SCM-4.

To eliminate the influence between adjacent nanoindentation points, the space was selected as 10 μm. In addition, the indentations of the unhydrated cement particles with a modulus greater than 50 GPa and the indentations of the porosity with a modulus in the range of 0–8 GPa were removed; the two types of indentations can be easily identified based on the load–penetration depth curve. Figure 9a,b shows the nanoindentation experimental results of SCM-1 and SCM-4. The nanoindentation data plotted on the same abscissa were averaged and connected, thus obtaining the trend more intuitively. The ITZ between the aggregate and the cement matrix can be distinguished from the change in the elastic modulus. For the natural river sand aggregate–cement matrix system, the aggregate distribution is in the range of 0–20 μm, and the elastic modulus at the nanoindentation points is greater than 60 GPa. From a test region ranging from 20 to 40 μm for SCM-1, there is a significant drop in the elastic modulus, which is the minimum value in the entire nanoindentation test region. Therefore, this part is inferred as the ITZ between the aggregate and the cement matrix. Between 20 and 40 μm for SCM-1, the elastic modulus range is 10–40 GPa. Similarly, for the coral sand aggregate–cement matrix system, the aggregate distribution is in the range of 0–20 μm, and the elastic modulus at the nanoindentation points is greater than 50 GPa. The elastic modulus between 20 and 40 μm also shows a significant drop for SCM-4, and its elastic modulus range is 9–20 GPa. According to the elastic modulus of the ITZ under different aggregates, the elastic modulus of the ITZ of the coral mortar is lower than that of the natural river sand mortar, consistent with the experimental results of the DMA. The storage modulus of the coral mortar is less than that of the natural river sand mortar. A significant number of ITZs are observed in the cement-based material between the aggregate and the cement matrix. The mechanical properties of the ITZ influence the mechanical properties of the samples. As mentioned earlier, the storage modulus of the SCM can be characterized in terms of the stiffness, which is proportional to the elastic modulus. In the process of energy transfer, the ITZ acts a propagation medium. An ITZ with a lower modulus of elasticity has a lower stiffness and a negative effect on energy storage. Therefore, the multiple ITZs of the coral mortar significantly reduce the storage modulus.

### 3.6. Effect of Microstructure on the Dynamic Mechanical Behavior of Seawater Coral Mortar

**Figure 10:**
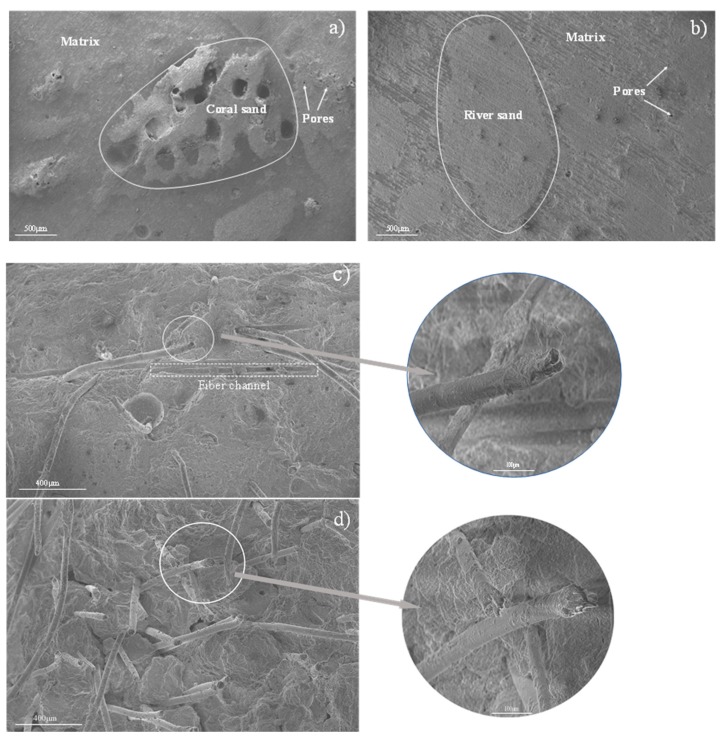
Microstructure of the mortar: (**a**) SCM-1; (**b**) SCM-4; (**c**) friction-damaged microstructure of PVA fiber ends; (**d**) fracture-damaged microstructure of PVA fiber ends.

Figure 10a,b shows the microstructures of the SCM samples with and without coral sand cured for 28 days. Among the samples, the reference sample (SCM-1) exhibits the more obvious ITZ between the river sand and the cement matrix, as shown in Figure 10a. Based on the microstructural morphology of the coral sand, the coral sand aggregate and matrix in the sample can be distinguished in Figure 10b. Due to the irregular surface shape of the coral sand, the ITZ between the coral sand and the matrix is inconspicuous. With the increase in the coral sand replacement rate, the cement matrix became less compact owing to the pores of the coral sand itself in the hardened mortar. Due to the existence of such multi-structures between the coral sand and the cement matrix, the density of the samples was insufficient, and the storage modulus was lower than that of the sample without coral sand.

Figure 10c,d shows the microstructure of the PVA fiber attached to the mortar. A tight connection between the cement matrix and the PVA fiber can be observed, owing to the hydrophilicity of the PVA fiber. Figure 10c,d shows the SEM images of the fiber ends after being subjected to a dynamic load. The SEM results indicate that the failure morphology of the PVA fiber ends in the cement matrix under dynamic loading can be mainly divided into two types. The width of the fiber channel was measured by SEM and compared with the diameter of the PVA fiber to confirm that the fiber channel was formed because of the relative movement between the PVA fiber and the matrix. Figure 10c shows the frictional damage caused by the PVA fiber under dynamic loading. Another failure morphology was the direct fracture of the PVA fiber under external loads, as shown in Figure 10d. For the PVA fiber-reinforced samples, energy was consumed during the friction generated by the fiber and the matrix and the breakage of the fiber under the action of the external dynamic load. This validates the DMA results, which showed that the loss factor of the PVA fiber-reinforced samples is higher than that of the sample without the PVA fiber.

### 3.7. Mechanisms on Dynamic Mechanical Properties

The effects of varying coral aggregates replacement rates and PVA fiber contents on dynamic mechanical properties were discussed. The combination of SNT experiments and SEM experiments testify the reinforcing mechanism of dynamic mechanical properties.

**Figure 11:**
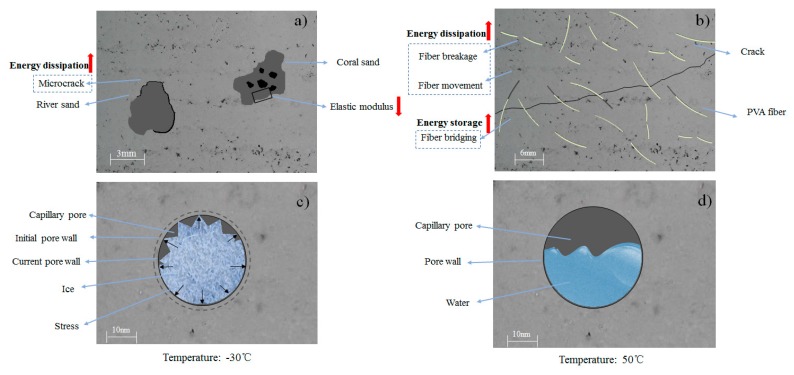
Schematic diagram of the reinforcing mechanism on dynamic mechanical properties of marine mortar: (**a**) Coral sand aggregate replacement; (**b**) PVA fiber reinforcement; (**c**) after cooling process (−30 °C < T < 0 °C); (**d**) after heating process (0 °C < T < 50 °C).

**Aggregate replacement:** The storage modulus is usually inversely proportional to the amount of deformation, which means that the matrix containing coral sand is more likely to deform under dynamic loading. In other words, the samples containing coral sand are more vulnerable to brittle failure under dynamic loading, and the ability to store energy is less. This is probably because the ITZ of the coral aggregates has a weaker energy storage capacity and a lower elastic modulus, as shown in Figure 11a.

The interface formed between the coral sand and the cement matrix is more compact than that formed between natural river sand and the cement matrix; this is attributed to the porosity and irregular shape of the coral sand aggregates. However, the microcracks formed between the river sand and the cement matrix can convert energy into mechanical energy via the vibration of the aggregates, thus promoting energy consumption, as shown in Figure 11a. Under cycling loading, the coral sand mortar loses it carrying capacity faster than the river sand mortar. When the coral sand mortar finally loses the carrying capacity, it accumulates less damage than the river sand mortar. 

**Fiber reinforcement:** This can be attributed to the fact that the fibers hinder the further development of cracks and that the stiffness of the cement matrix is greater than that of the reference sample SCM-4, as shown in Figure 11b. Thus, the addition of PVA fiber has a positive effect on the SCM storage modulus.

Energy may be partly dissipated at the vicinity of the fiber ends in the form of micro-plastic strain, and the inherent stress concentration in the cement–matrix could be released [40]. This means that the PVA fiber in marine mortar is damaged under the dynamic loading. A portion of the energy is consumed during the elastic deformation and breakage of the PVA fiber; this shows that the fiber-added marine mortar consumes more energy than the marine mortar without the fiber, as shown in Figure 11b. The relative displacement between the PVA fiber and the cement matrix produces friction that consumes a part of the energy during the fiber pull-out process under the load. The presence of fibers increases the load carrying capacity of the mortar prior to initial cracking, resulting in a continuous uniform gradient of the stress field [41]. Moreover, they help increase the toughness of the cement matrix and the load-bearing capacity after cracking. A higher toughness of the cement matrix means higher deformability. The stress concentration is effectively mitigated, and the shock resistance performance is improved by absorbing deformation energy and converting it into potential energy [42].

**Temperature change:**Figure 11c,d shows the mechanism. When the internal temperature of the mortar is below 0 °C, the pressure between the capillary pore walls due to temperature contraction and the ice expansion lead to excessive stress in the mortar. The microstructure of the cement matrix is damaged by the excessive stress. Therefore, the loss factor is minimum at −30 °C. In addition, when the internal temperature of the mortar is above 0 °C, the ice in the capillary pores gradually melts, so the stress mitigated due to ice expansion and the medium in which the energy propagates inside the cement matrix changes. This could explain the rapid increase in the loss energy between 0 and 50 °C.

## 4. Conclusions

This paper reports the workability, mechanical properties, and dynamic mechanical behavior of seawater coral mortar (SCM) containing varying coral aggregate replacements and PVA fiber contents at a constant water–cement ratio of 0.4. The following conclusions can be drawn from the experimental results:

(1) Compared with the reference sample SCM-1, the slump flow expansion of SCM-4 was reduced by 19.2%, which was attributed to the rough and multi-angled surface of coral aggregates. With the incorporation of 1 wt.% PVA fiber, the workability of SCM-4 decreased by 13.0%.

(2) Compared with the reference sample SCM-1, the incorporation of coral aggregates (100% replacement rate) reduced the compressive and flexural strengths cured for 28 days by 15.9 and 9.0%, respectively. The incorporation of PVA fibers led to a slight increase in the compressive strength. With the addition of 1 wt.% PVA fiber, the flexural strengths of the samples cured for 28 days increased by 32.0% compared with that of the reference sample SCM-4.

(3) The storage modulus and loss factor of the SCMs decreased with increasing coral sand aggregate replacement rates in the temperature range of −30 to 50 °C. Notably, the elastic modulus of the ITZ of coral sand was lower than that of the ITZ of natural river sand. With the addition of 1 wt.% PVA fiber, the storage modulus and loss factors of the SCMs at a frequency of 0.5Hz improved by 151.9% and 73.3%.

(4) The mechanisms of the decreased storage modulus of the SCM-4 can be attributed to the lower elastic modulus of the ITZ between the coral sand aggregates and the cement matrix. The microcracks formed between the river sand and the cement matrix can promote energy consumption. Moreover, the fiber reinforcement mechanisms in storage modulus is attributed to the fibers hindered the further development of cracks and that the stiffness of the cement matrix was greater than that of the reference sample SCM-4. The enhancement in the energy dissipating behavior was attributed to the improved energy consumption of the SCM by generating multiple cracks and fiber deformation under dynamic loading.

(5) Between −30 and 0 °C, the water inside the capillary pores of the mortar may have frozen and filled the capillary pores, resulting in a stress between the contracted pore walls and ice expansion that destroyed the matrix and reduced the energy consumption. From 0 to 50 °C, the frozen water in the pores gradually melted, and the presence of air in the pores altered the propagation medium for energy dissipation. 

## Figures and Tables

**Table 1:** Chemical compositions of the cement and fly ash used in this study (wt.%).

Ingredient	CaO	SiO_2_	Al_2_O_3_	Fe_2_O_3_	MgO	SO_3_	K_2_O	Na_2_O	LOI
Cement	64.42	20.52	5.62	3.88	2.11	2.10	0.28	0.20	0.87
Fly ash	7.08	43.34	25.84	5.46	1.17	2.37	1.05	1.13	3.79

**Table 2:** Ionic compositions of the seawater taken from the South China Sea.

Ion	K^+^	Na^+^	Ca^+^	Mg^+^	Cl^−^	SO_4_^2−^
Concentration, g/L	0.56	16.00	0.50	2.70	26.00	4.70

**Table 3:** Physical properties of coral sand and natural river sand.

Material Characteristics	Coral Sand	Natural River Sand
Bulk density (kg/m^3^)	1280	1490
Apparent density (kg/m^3^)	2740	2630
Water absorption (%)	3.4	0.55

**Table 4:** Physical properties of polyvinyl alcohol (PVA) fiber.

Density (g/cm^3^)	Tensile Strength (MPa)	Elastic Modulus (GPa)	Limited Elongation (%)	Length (mm)	Diameter (μm)
1.3	1620	42.8	7.8	12	40

**Table 5:** Mixture proportions of the mortar used in this study.

Sample	Cement (g)	Fly Ash (g)	Water (g)	W/C	Sand (g)		Fiber (volume%)	HRWR (g)
					Standard Sand	Coral Sand		
SCM-1	727	182	364	0.4	1000	0	0	1.6
SCM-2	727	182	364	0.4	700	300	0	1.6
SCM-3	727	182	364	0.4	400	600	0	1.6
SCM-4	727	182	364	0.4	0	1000	0	1.6
SCM-5	727	182	364	0.4	0	1000	0.25	1.6
SCM-6	727	182	364	0.4	0	1000	0.5	1.6
SCM-7	727	182	364	0.4	0	1000	1	1.6
